# Regional variation in tolvaptan prescribing across England: national data and retrospective evaluation from an expert centre

**DOI:** 10.1093/ckj/sfac190

**Published:** 2022-08-26

**Authors:** Jiehan Chong, Tess Harris, Albert C M Ong

**Affiliations:** Academic Nephrology Unit, Department of Infection, Immunity, and Cardiovascular Disease, Medical School, University of Sheffield, Sheffield, UK; Sheffield Kidney Institute, Sheffield Teaching Hospitals NHS Foundation Trust, Sheffield, UK; Leeds Institute of Cardiovascular and Metabolic Medicine, Medical School, University of Leeds, Leeds, UK; PKD Charity, London, UK; Academic Nephrology Unit, Department of Infection, Immunity, and Cardiovascular Disease, Medical School, University of Sheffield, Sheffield, UK; Sheffield Kidney Institute, Sheffield Teaching Hospitals NHS Foundation Trust, Sheffield, UK

**Keywords:** autosomal dominant polycystic kidney disease, patient selection, PKD1, PKD2, prognostic risk assessment, tolvaptan, total kidney volume

## Abstract

**Background:**

Tolvaptan, a vasopressin V2 receptor antagonist, was approved in 2015 by the UK National Institute for Health and Care Excellence for use in patients with autosomal dominant polycystic kidney disease (ADPKD) and rapid disease progression. Simultaneous guidance was issued by the UK Kidney Association (UKKA) to facilitate national implementation.

**Methods:**

Data on tolvaptan prescribing in England was obtained through the National Health Service (NHS) Digital, a national survey of all 77 adult kidney units, and the implementation of UKKA guidance was evaluated at an expert PKD centre.

**Results:**

A regional variation of up to 4-fold for tolvaptan prescribing in England was found. Despite most kidney units following UKKA guidance, centre-based estimates of eligible or treated patient numbers were highly variable. Retrospective evaluation at an expert PKD centre revealed that in a cohort demonstrating rapid estimated glomerular filtration rate (eGFR) decline, 14% would not be eligible for tolvaptan by Mayo imaging classification and more than half (57%) would not be eligible by Predicting Renal Outcome in Polycystic Kidney Disease score. The 3-year discontinuation rate was higher than expected (56%), the majority (70%) due to aquaretic symptoms. In patients taking tolvaptan for at least 2 years, 81% showed a reduction in the rate of eGFR decline compared with baseline, with earlier disease associated with positive treatment response.

**Conclusion:**

Real-world data have revealed a much higher regional variation in tolvaptan prescribing for ADPKD in England than expected. We propose further investigation into the factors responsible for this variation.

## Graphical Abstract

**Graphical Abstract:**
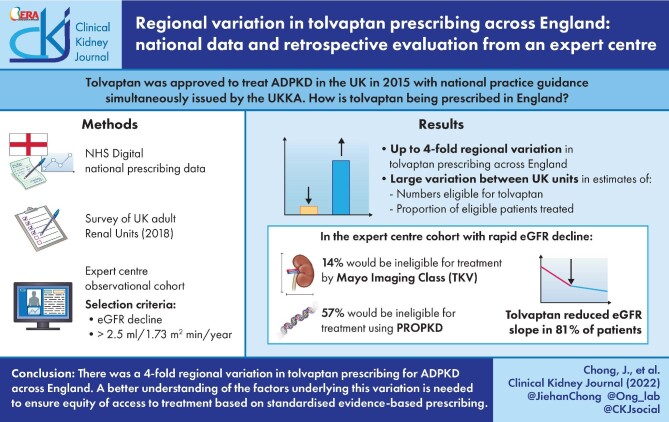

## INTRODUCTION

Autosomal dominant polycystic kidney disease (ADPKD) is the most common inherited cause of kidney disease [1, 2] and accounts for 10.4% of prevalent patients on renal replacement therapy (RRT) in the UK [3]. Until recently, clinical management of ADPKD has consisted of general chronic kidney disease (CKD) management, such as blood pressure control, combined with symptomatic management of disease-specific complications [2, 4].

In 2015, tolvaptan, a vasopressin V2 receptor antagonist was approved by the European Medicines Agency for use in ADPKD patients with ‘evidence of rapid disease progression’, following the pivotal Tolvaptan Phase 3 Efficacy and Safety Study in Autosomal Dominant Polycystic Kidney Disease (TEMPO 3:4) trial [5]. The major side effects were aquaresis and idiosyncratic hepatotoxicity that led to treatment discontinuation in 23% of the treated group. Following a second phase 3 trial, Replicating Evidence of Preserved Renal Function: An Investigation of Tolvaptan Safety and Efficacy in ADPKD (REPRISE) [6], in 2018, showing similarly beneficial effects in later-stage disease, the US Food and Drug Administration (FDA) approved tolvaptan for ADPKD in the USA. Long-term studies have suggested a sustained benefit from tolvaptan therapy over 5 years [7, 8].

The National Institute for Health and Care Excellence (NICE) similarly approved tolvaptan for use in England [9]. Following this, the UK Kidney Association (UKKA), previously the Renal Association, issued a commentary on the NICE decision [10], to facilitate national implementation. As the NICE decision and UKKA commentary were published prior to the REPRISE study, they do not include patients with an estimated glomerular filtration rate (eGFR) <30 ml/min/1.73 m^2^, despite evidence now supporting this [6].

Tolvaptan has now been used to treat ADPKD in the UK for >5 years. Nonetheless, the extent to which it is being prescribed, barriers to its adoption and real-world evidence of its safety, tolerability and efficacy have not been previously reported. In this article we report current tolvaptan prescription practices in the UK using a combination of publicly available prescribing data, a national survey of clinical practice and detailed follow-up data from an expert centre applying RA guidance.

## MATERIALS AND METHODS

### National and regional data on prescribing in England

National and regional data on tolvaptan prescription were obtained from NHS Digital [11] and analysed in Python 3.8.5 (https://www.python.org/), using the pandas 1.2.2 (https://pandas.pydata.org/pandas-docs/stable/index.html) [12] and Matplotlib 3.2.2 (https://matplotlib.org/3.2.2/#) [13] libraries. Tolvaptan prescription data were obtained as defined daily doses (DDD) per 100 000 population. The number treated was calculated using the World Health Organization definition for tolvaptan DDD (30 mg) and assumed an average dose of 95 mg/person/day, the average tolvaptan dose in published trials [5, 6].

### National survey of tolvaptan use in all UK adult kidney units

A national survey of tolvaptan use was developed using the QuestionPro platform and distributed via the UK Renal Clinical Directors’ Network in 2018. Survey questions are outlined in the Supplementary material (Detailed Methods). The results were analysed in Excel (Microsoft, Redmond, WA, USA). Not all respondents answered every question; in determining the proportion of responses to each question, the denominator was the number of responses received for that particular question.

### Retrospective evaluation of tolvaptan use at an expert centre based on RA guidance

A retrospective analysis was performed on a cohort of patients prescribed tolvaptan at an expert PKD centre (Sheffield) over a 3-year period (2017–2020).

eGFR was calculated using the Chronic Kidney Disease Epidemiology Collaboration (CKD-EPI) equation [14]. The rate of eGFR decline was calculated by linear regression, with measurements taken during the dose titration period excluded from on-treatment calculations to account for the initial eGFR decrease associated with starting tolvaptan therapy. Baseline measurements of total kidney volume by magnetic resonance imaging (MRI-TKV) and genotyping were performed to calculate prognostic scores for disease progression [15, 16]. MRI-TKV was calculated by manual segmentation [17]. Comprehensive clinical data were prospectively collected at baseline and at each follow-up visit (Supplementary Table 1). The data were analysed in Python 3.8.10 using pandas 1.3.2, SciPy 1.7.1 (https://docs.scipy.org/doc/scipy/index.html) [18] and Matplotlib 3.4.3.

Detailed analysis methods are outlined in the Supplementary material.

## RESULTS

### Regional variation in tolvaptan prescription and practice

**Figure 1:**
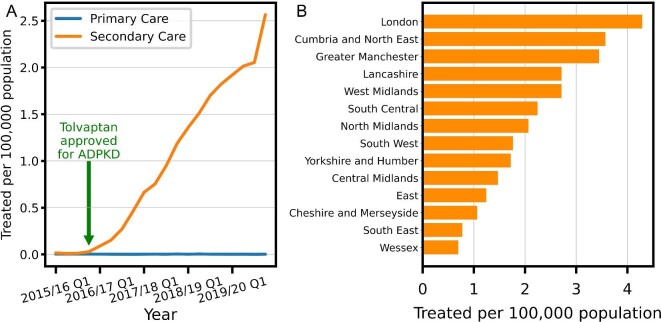
Tolvaptan use in England. **(A)** Quarterly total tolvaptan use in England for all indications. **(B)** Tolvaptan use from 1 January to 31 March 2020 in different English regions.

To understand the extent of tolvaptan prescription in England, we analysed national tolvaptan prescription data for England from 2015 to 2020. Following NICE approval in late 2015, per-capita tolvaptan prescription in England has increased steadily each quarter (Figure 1A). Prescriptions at the end of 2020 are >700-fold higher than before tolvaptan was approved for ADPKD. As there have been no new clinical indications for tolvaptan over this period, we can safely assume that almost all tolvaptan currently prescribed in England is for ADPKD. Using an average daily dose of 95 mg for ADPKD patients on tolvaptan (consistent with the lower end of average doses in published trials [5, 6]), the total amount prescribed nationwide would treat 1444 patients as of April 2020. With an estimated point prevalence of 1 in 2500 [19] and the population of England being 56.5 million [20], approximately 6% of prevalent ADPKD patients in England are being treated with tolvaptan. Taking a more conservative estimate of the average dose (60 mg), 2273 patients (10%) would be treated. Surprisingly, we observed striking regional variations in tolvaptan usage, with a 4-fold difference between areas with the highest and lowest usage (Figure 1B).

**Figure 2:**
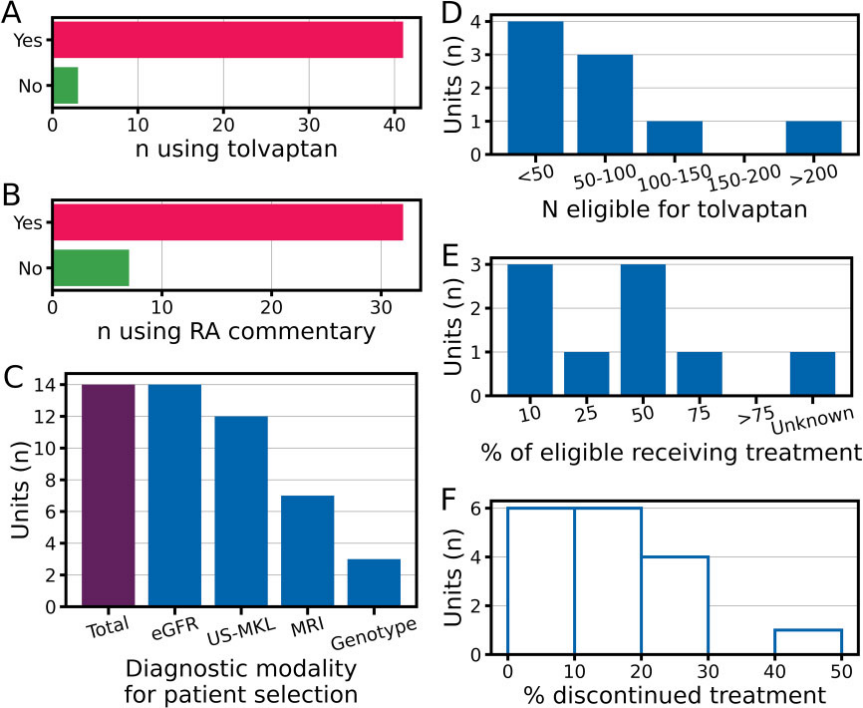
Results of a national survey of UK adult kidney units regarding tolvaptan prescribing practices. **(A)** Bar chart of number of units using tolvaptan. **(B)** Bar chart of number of units using the RA commentary. **(C)** Bar chart of number of units using each diagnostic modality for treatment selection. **(D)** Bar chart of number of patients eligible for tolvaptan. **(E)** Bar chart of proportion of patients eligible for tolvaptan who are receiving treatment. **(F)** Histogram of proportion of patients discontinuing treatment at each unit.

To investigate tolvaptan prescribing practice and UKKA guidance application in different renal units, we conducted a survey of all UK renal units in 2018, 3 years after NICE approval. Of the 77 adult UK renal units surveyed, 44 responses were returned and 93% (41/44) of respondents reported using tolvaptan to treat ADPKD (Figure 2A). Reasons given for not using tolvaptan were a lack of suitable patients, side effect profile and lack of evidence of sustained benefit. A total of 82% (32/39) reported using the UKKA commentary to guide tolvaptan use (Figure 2B). Reasons cited for not using the commentary were the use of alternative guidance (European Renal Association or local alternative) and the inability to measure MRI-TKV.

All units used eGFR to assess for treatment eligibility and most (12/14) also used ultrasound mean kidney length (US-MKL). Only half reported using MRI-TKV and a minority (3/14) used genotype (Figure 2C). There was a huge variation in the responses to questions regarding the number of patients in each unit eligible for tolvaptan, the proportion of eligible patients taking tolvaptan and the number of treated patients who had stopped tolvaptan (Figure 2D–F).

Overall, the RA survey confirmed that tolvaptan was being prescribed across the UK employing a multidisciplinary approach but differences in local practice affecting candidate identification or recruitment for treatment and retention on treatment might contribute to the observed regional variations in tolvaptan prescribing.

### Application of UKKA guidance at an expert PKD centre

To identify elements of clinical practice affecting candidate identification, recruitment to treatment and side-effect tolerance, we evaluated experience at an expert PKD centre rigorously applying the UKKA commentary between 2017 and 2020 (Sheffield). The baseline characteristics of the cohort are shown in Table 1. The centre employed comprehensive screening of all ADPKD patients not on renal replacement therapy for eGFR decline >2.5 ml/min/1.73 m^2^/year over 5 years, in accordance with the UKKA guidance [10], repeated every 6 months. Using this approach, >360 patients are screened every 6 months, and over 4 years >100 candidates have been identified, resulting in 43 patients started on tolvaptan.

**Table 1:** Baseline characteristics of tolvaptan-treated expert centre cohort (*N* = 43).

Characteristics	*n* (%)			
Male	18 (42)			
Female	25 (58)			
White ethnicity	41 (95)			
Asian	1 (2)			
Other ethnicity	1 (2)			
Total	43 (100)			
	
	Average^a^	Maximum	Minimum	SD
Age (years)	44	61	24	9.68
eGFR (ml/min/1.73 m^2^)^b^	56	99	27	20.02
eGFR decline (ml/min/1.73 m^2^/year)	−6.1	−2.0	−17.3	3.47
TKV (ml)^c^	1612	4797	504	1145

^a^Mean except for TKV, where median is shown.

^b^Calculated by the CKD-EPI equation including race.

^c^Calculated by planimetry with manual segmentation.

All patients started treatment on 45/15 mg/day, and this was increased on a monthly basis until 90/30 mg/day or the highest tolerated dose was reached. At the point of analysis, 32/43 patients had completed dose titration. Of these, 19/32 (59%) had reached a dose of 90/30 mg, 7/32 (22%) were taking 60/30 mg and 6/32 (19%) were on 45/15 mg at the end of the dose titration period.

The performances of imaging and genotype risk scores in identifying patients with rapid eGFR decline were compared. The UKKA guidance distinguishes patients with either ‘evidence of rapid decline’ in the form of a rapid eGFR decline and those with ‘risk of rapid decline’, defined primarily using the Mayo imaging classification (MIC) based on height-adjusted TKV or the Predicting Renal Outcome in Polycystic Kidney Disease (PROPKD) score [10]. To explore the sensitivity and specificity of ‘risk’ criteria in patients with rapid eGFR decline, we analysed the distribution of these parameters around their recommended cut-offs for eligibility (Table 2).

**Table 2:** Distribution of MIC, US-MKL and PROPKD scores above and below eligibility cut-offs in an expert centre cohort (*N* = 43) assessed under current UKKA guidance.

Assessment modality (cut-off)	Above cut-off, *n* (%)	Below cut-off, *n* (%)
MIC1B	37 (86)	6 (14)
US-MKL (16.5 cm)	14 (74)	5 (26)
PROPKD score (6)	13 (43)	17 (57)

Using MRI-TKV, most patients fell into the expected group associated with rapid disease progression (MIC1C–E), although a proportion of patients (14%) were classified into MIC1B. Current guidance recommends a US-MKL ≥16.5 cm as a cut-off to select patients for further TKV measurement by cross-sectional imaging [21]. A total of 19 patients in this cohort had an ultrasound scan within 1  year of commencing tolvaptan. Notably, 5 (26%) had a US-MKL <16.5 cm and would therefore have been excluded.

A total of 25 patients had a *PKD1* truncating mutation either detected in the patient or as a known familial mutation, 8 patients had a non-truncating *PKD1* mutation, 5 had a *PKD2* mutation and 1 had no mutation detected. The PROPKD score was calculated in 30 patients with available information. Surprisingly, 17 (57%) had a PROPKD score <6 and would not have been eligible (Supplementary Figure 1). Selecting purely for the presence of a truncating PKD1 mutation more accurately classifies this cohort (Supplementary Table 2), excluding a smaller proportion (36%) [15]. Interestingly, of the six patients with MIC1B, 5 had *PKD1* truncating mutations but only 2 had a PROPKD score >6.

### Treatment discontinuation is primarily due to aquaresis

**Figure 3:**
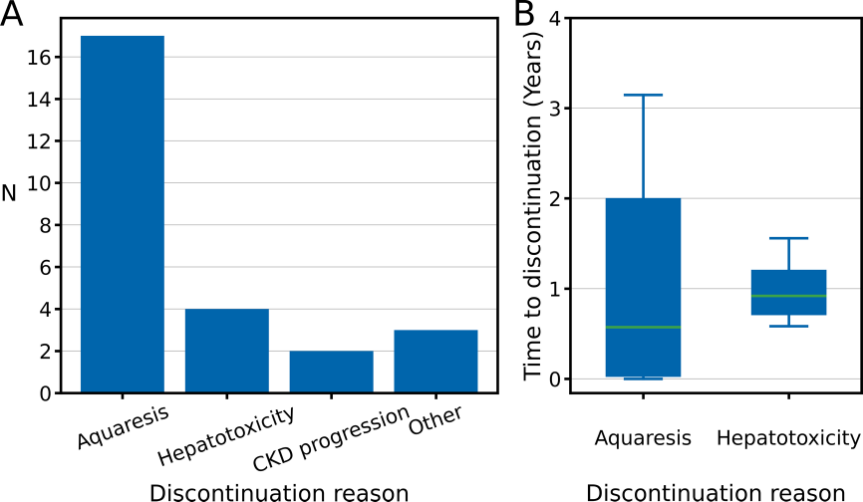
Treatment discontinuation is high and primarily due to aquaretic symptoms. **(A)** Number of patients with each reason for discontinuation. **(B)** Boxplot of time to treatment discontinuation by aquaresis and hepatotoxicity. Boxes represent quartiles 1–3. Whiskers represent maximum and minimum values.

To investigate factors influencing tolvaptan tolerance, we analysed the reasons for treatment discontinuation in this cohort. Of 43 patients who commenced tolvaptan, 14 (32%) discontinued treatment within the first year and 24 (56%) discontinued by 3 years. Most patients [17/24 (70%)] stopped treatment due to aquaretic symptoms (Figure 3A). At the point of analysis, only 18 patients remained on tolvaptan. Assuming a point prevalence of 1 in 2500, this represents only 4% of prevalent ADPKD patients in the centre's catchment area.

While discontinuation due to aquaresis occurs mainly within the first year, some patients persisted for >3 years before stopping treatment (Figure 3B). The second most common reason for treatment cessation was hepatotoxicity [4/24 (17%)], which could occur up to 18 months after commencing tolvaptan (Figure 3B).

### Factors influencing urine volume

Given the importance of aquaresis as a limiting factor for treatment tolerance, we analysed clinical data on 24-hour urine volume, sodium excretion and osmolality. There was no significant difference in pre-treatment spot urine osmolality between dosage groups (*P* = .66). After completing dose titration there was no significant difference between dosage groups in 24-hour urine volume (*P* = .74) or spot urine osmolality (*P* = .79). The mean 24-hour urine volume was 7.4 l, but there was substantial variation, especially at the highest dose (120 mg/day), with some patients exceeding >12 l/day (Supplementary Figure 2). The 24-hour urine sodium excretion showed a linear correlation with 24-hour urine volume, confirming that urine volume is closely correlated with dietary sodium intake (Supplementary Figure 3).

### Predictors of long-term individual response to tolvaptan

**Figure 4:**
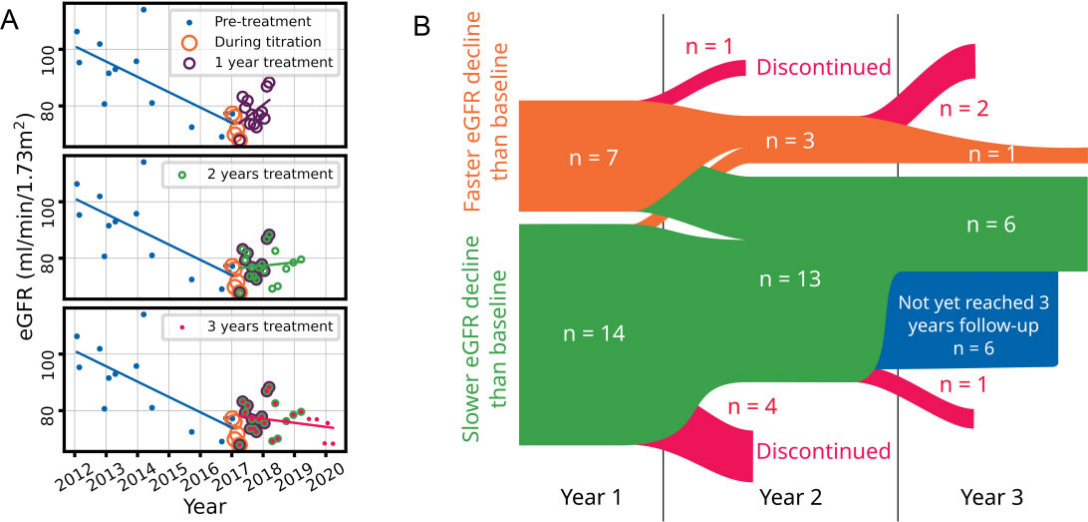
Long-term individual responses to tolvaptan over 3 years on treatment. **(A)** Scatter plots of eGFR over time in a single patient, illustrating how the regression slope can change dramatically over time. **(B)** Flow diagram of response to treatment over 1, 2 and 3 years. Orange limbs represent patients in whom tolvaptan treatment was associated with a faster eGFR decline compared with baseline over the associated period. Green limbs represent patients in whom tolvaptan was associated with a slower eGFR decline compared with baseline. Red limbs represent patients discontinuing treatment. The thickness of each limb is proportional to the number of patients represented for that period.

Given the poor tolerability of tolvaptan in clinical practice, we analysed individual treatment responses to determine where treatment could be safely stopped. The rate of eGFR decline over 1, 2 and 3 years on treatment was compared with the pre-treatment rate of eGFR decline (Supplementary Table 3). This is illustrated for a single patient in Figure 4A. A slower eGFR decline on treatment compared with baseline was considered a positive treatment response, whereas a faster eGFR decline was considered a negative response. There was a statistically significant reduction in the rate of eGFR decline over 2 and 3 years (*P* = .008 and .03, respectively), but not at 1 year (*P* = .18), compared with baseline. A total of 33% (7/21) of patients had a negative response over the first year (Figure 4B). 57% (4/7) of negative responders after the first year had a positive response at 2 years and 81% (13/16) of patients had a positive response at 2 years, with no further change after 2 years.

The mean relative reduction in annual eGFR decline was the same over 1, 2 and 3 years (*P* = .66). However, the standard deviation (SD) decreased as the duration of assessment increased, despite the decreasing sample size (Supplementary Table 4). As treatment response in this cohort was stable after 2 years, we sought predictors of 2-year treatment response by linear regression against factors previously associated with tolvaptan response [22, 23] (Supplementary Table 5). Individual treatment response with baseline eGFR, initial urine osmolality and osmolality change during dose titration were associated with positive treatment response (*P* = .05, .03 and .05 respectively) but are not useful for individual response prediction due to weak correlations (*r* = −0.48, −0.55 and 0.51, respectively).

## DISCUSSION

The major finding of this study is the wide regional variation of tolvaptan prescribing for ADPKD in England. We have attributed this variation primarily to differences in candidate identification, retention and recruitment, but could there also be differences in regional prevalence? There is substantial ethnic variation between regions [24] and ADPKD prevalence elsewhere in the world does vary with ethnicity [25]. However, the reported variation in prevalence between different ethnic groups is <2-fold [25], far smaller than the 4-fold difference in tolvaptan prescription between regions.

It is notable that despite comprehensive candidate identification, only 4% of prevalent ADPKD patients in our centre's catchment area are being treated with tolvaptan—less than the national estimated average of 6%. The highest-prescribing units may employ more effective strategies for patient recruitment and retention. Alternatively, they could have included patients who are not strictly eligible. For units with low prescribing rates, better candidate identification should increase prescribing.

How should eligible candidates be identified? The rate of eGFR decline was the major treatment outcome in ADPKD trials, with TKV acting as a surrogate measure [16]. The number of patients with prognostically good MIC, US-MKL and PROPKD scores seems surprising in a cohort of patients proven to have rapid eGFR progression. However, this is consistent with the performance characteristics of these metrics as described in the original papers. MIC is based on a longitudinal mixed effects model in which there is overlap between the confidence intervals of the time interaction terms, most notably between Class 1B and 1C [16]. Second, although a PROPKD score >6 strongly predicts ESRD before age 60 years with a positive predictive value of 90.9%, the negative predictive value is only 57.3%. Even using a lower threshold score of 3, the negative predictive value was 81.4% in the derivation cohort [15]. Third, US-MKL has a reported sensitivity and specificity of 82.9% and 80.8%, respectively, for predicting CKD progression (onset of CKD stage 3 within 8 years) in ADPKD [21]. While the survey suggests that MRI-TKV availability is an issue, we suspect the limitation is TKV measurement rather than obtaining scans. The availability of newer semi-automated and automated methods for TKV measurement will make this issue irrelevant in the future [17, 26]. In the interim, the ellipsoid equation requires only two length measurements per kidney, has reasonable performance compared with more sophisticated methods [27] and in fact was used to derive the MIC [16].

Our results suggest that in a cohort preselected for rapid eGFR decline, there are limited gains from including TKV or PROPKD scores. MIC would have excluded 14% of this cohort with proven rapid eGFR decline and the PROPKD score would have excluded more than half. As most of the patients with MIC1B also had PROPKD scores <6, combining TKV and PROPKD does not dramatically improve on the performance of TKV alone [15].

To accurately establish the rate of eGFR decline, sufficient follow-up duration is key. The current UKKA guidance accepts a decline of 5 ml/min/1.73 m^2^ over 1 year or 2.5 ml/min/1.73 m^2^ over 5 years. In this cohort, the SD of the change in eGFR decline decreases dramatically with an increasing length of follow-up (Table 3). This likely reflects the uncertainty of measurement for serum creatinine, and by extension eGFR, which is divided by an ever-larger denominator as the number of years assessed increases. In patients with CKD stage 1–2, variation between assays, or even within the same assay, easily exceeds the threshold for tolvaptan eligibility, even with the most accurate assay types [28]. Given this, the 5 ml/min/1.73 m^2^ over 1 year criterion in the current UKKA guidance cannot be evaluated with sufficient accuracy and should be removed.

How can we reduce treatment discontinuation? Our data agree with published data [5, 6, 29] suggesting that aquaretic symptoms are the main reason for stopping treatment. An association between dietary sodium intake and urine volume is well supported [30–32], but whether dietetic interventions to reduce sodium intake will reduce urine volume with tolvaptan has not been demonstrated. There may also be roles for thiazides and metformin [33, 34] in achieving this goal. It is important to recognize that there would still be significant aquaresis even with these measures [30, 31, 33, 34], and additional psychosocial interventions may be necessary to help patients cope with these changes and reduce treatment discontinuation.

The main weaknesses of this study are the incomplete responses to some questions in the national survey and the limited size of the longitudinal cohort. The latter may explain the discontinuation rate in this cohort, which is notably higher than previously reported in both clinical trials [5, 6] and real-world observational cohorts [29, 35] over comparable periods.

In summary, our results indicate that tolvaptan prescribing in England varies dramatically between regions. The reasons for this variation are likely to be centre-specific differences in candidate ascertainment, recruitment to treatment and side-effect management. Risk-based approaches miss a significant proportion of patients with rapid eGFR decline but may have a role in early disease. Reducing treatment discontinuation due to aquaresis could improve treatment rates, but further research is needed to identify the best ways to achieve this.

## Supplementary Material

sfac190_Supplemental_File

## Data Availability

The data underlying this article are available in the article and in its online supplementary material.
