# Supplementary material for: Regional variation in tolvaptan prescribing across England: national data and retrospective evaluation from an expert centre
Source: Clin Kidney J. 2022 Aug 26;16(1):61–8. doi: 10.1093/ckj/sfac190 (PMC9871855; doi:10.1093/ckj/sfac190)
Supplement: sfac190_Supplemental_File [file sfac190_supplemental_file.pdf]

Supplementary Table 1

Routinely collected clinical data and timing of collection

**Investigation**	**Frequency**
24-hour urine volume	Baseline then monthly during titration, then annually
24-hour urine osmolality	
Early first morning spot urine osmolality	Baseline then monthly for 18 months, then 3 monthly
Liver function tests	
MRI total kidney volume	Baseline
Genetic testing	Baseline

Supplementary Table 2

Distribution of mutation types

Mutation type	n
PKD1 truncating	23
PKD1 truncating in family (patient not tested)	2
PKD non-truncating	8
PKD2	5
No mutation detected	1

Supplementary Table 3

Rate of eGFR decline for each evaluation period and number of samples (n) associated with each rate calculation.

	Pre-treatment		Year 1 on treatment		Year 2 on treatment		Year 3 on treatment	
ID	n	Rate	n	Rate	n	Rate	n	Rate
1	16	-3.83	13	-0.37	19	-1.27	24	-1.57
2	9	-5.11	13	-2.42	18	-5.13		
3	20	-4.27	13	-2.74	18	-3.24	23	-2.78
4	14	-5.42	12	4.96	18	-0.29	23	-2.13
5	7	-3.96	21	3.24				
6	18	-1.98	10	-7.48	26	-5.83		
7	11	-5.12	16	-8.46	26	-7.79	32	-5.56
8	9	-7.24	14	-5.38	22	-4.48	27	-3.41
9	21	-17.33	13	-5.22				
10	10	-5.6	14	-6.46	22	-3.38	27	-2.45
11	15	-3.18	15	-5.28				
12	6	-10.67	13	7.16	23	-1.95	26	-3.19
13	7	-6.37	14	-5.16	22	-5.36		
14	9	-4.23	14	-13.38				
15	16	-10.23	14	-5.67	20	2.56		
16	6	-2.27	14	-7.79				
17	9	-3.35	14	-9.76	21	-2.31		
18	30	-6.54	14	4.94	20	0.39		
19	17	-4.6	14	-4.63	21	-1.83		
20	20	-9.54	18	-12.46	25	-5.18		
21	10	-2.95	13	-0.78	19	1.78		
22	6	-4.6	14	8.6				
23	4	-6.08	14	-3.72				

*Pre-treatment period is the 5 years prior to commencing tolvaptan. Rates are given in ml/min/1.73m^2.

Supplementary Table 4

Reduction in annual eGFR decline over years evaluated

Post-treatment period	n	Relative reduction in annual eGFR decline (%)	Std. dev. (%)
**1 year**	21	15*	141
**2 years**	16	40*	78
**3 years**	7	46*	24

**There was no significant difference in eGFR decline between evaluation periods (p = 0.66)*

Supplementary Table 5

Linear regression of potential factors influencing tolvaptan treatment response against 2-year % change in eGFR decline compared to baseline

	slope	r	p
**Baseline eGFR**	-1.81	-0.48	0.05
**Initial eGFR drop**	-0.24	-0.01	0.95
**TKV**	0.02	0.31	0.24
**Initial spot osmolality**	-0.33	-0.55	0.03
**Spot osmolality change during dose titration**	0.26	0.51	0.05

Supplementary Figure 1

Distribution of PROPKD scores

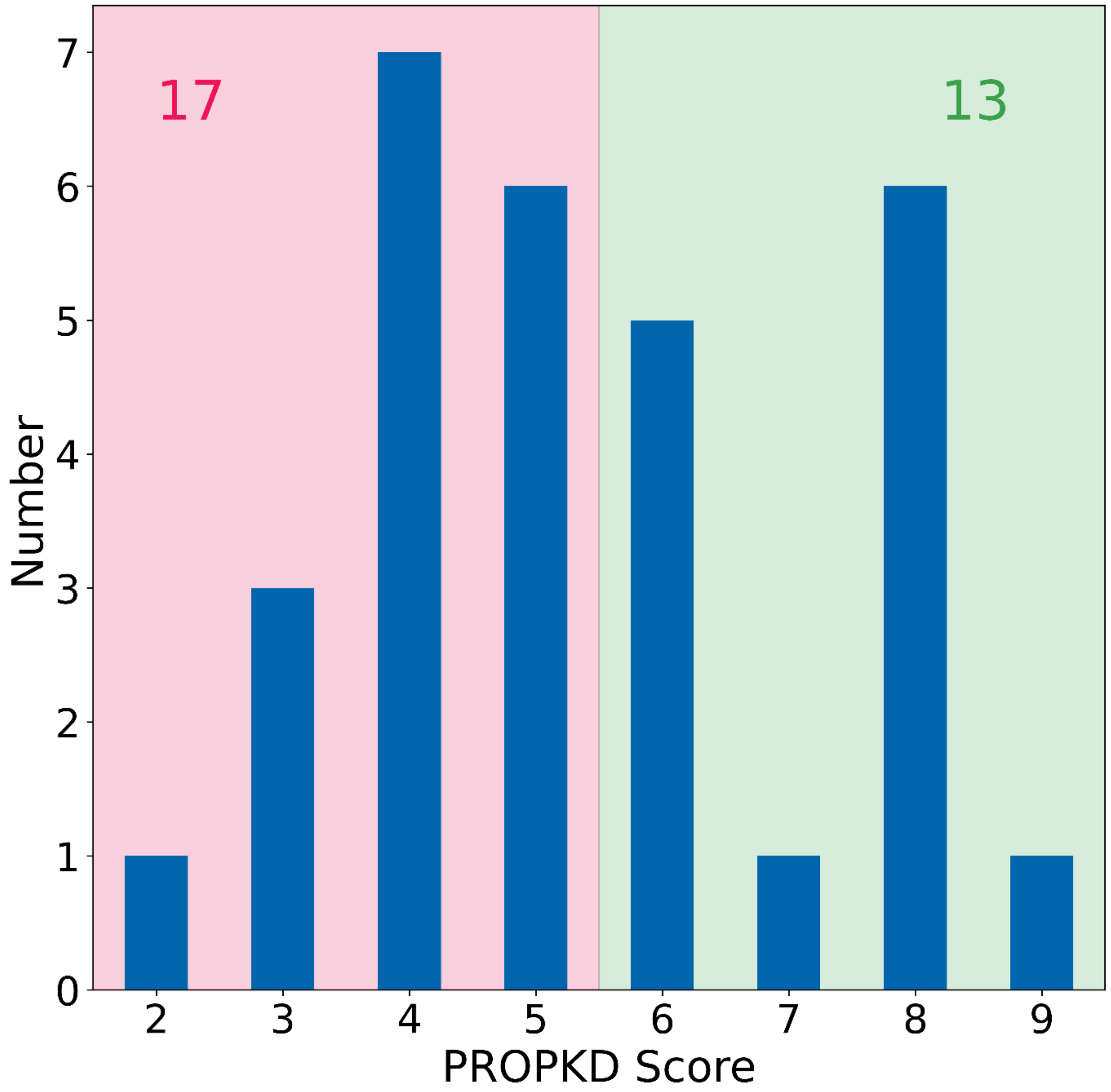

Supplementary Figure 2

Boxplot of 24hour urine volume, grouped by tolvaptan dose at end of titration period.

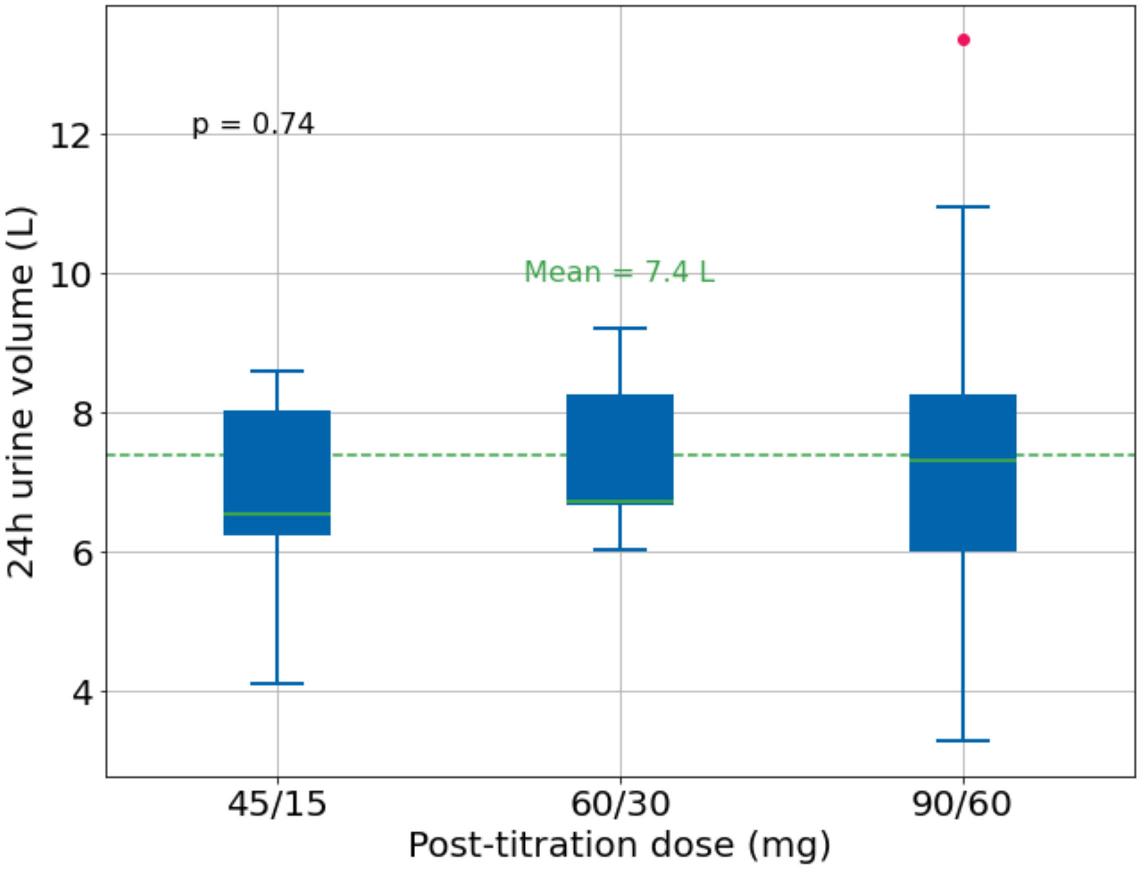

Supplementary Figure 3:

Scatter plot of 24-hour urine sodium against 24-hour urine volume on the same sample. Linear regression shown in dotted green line

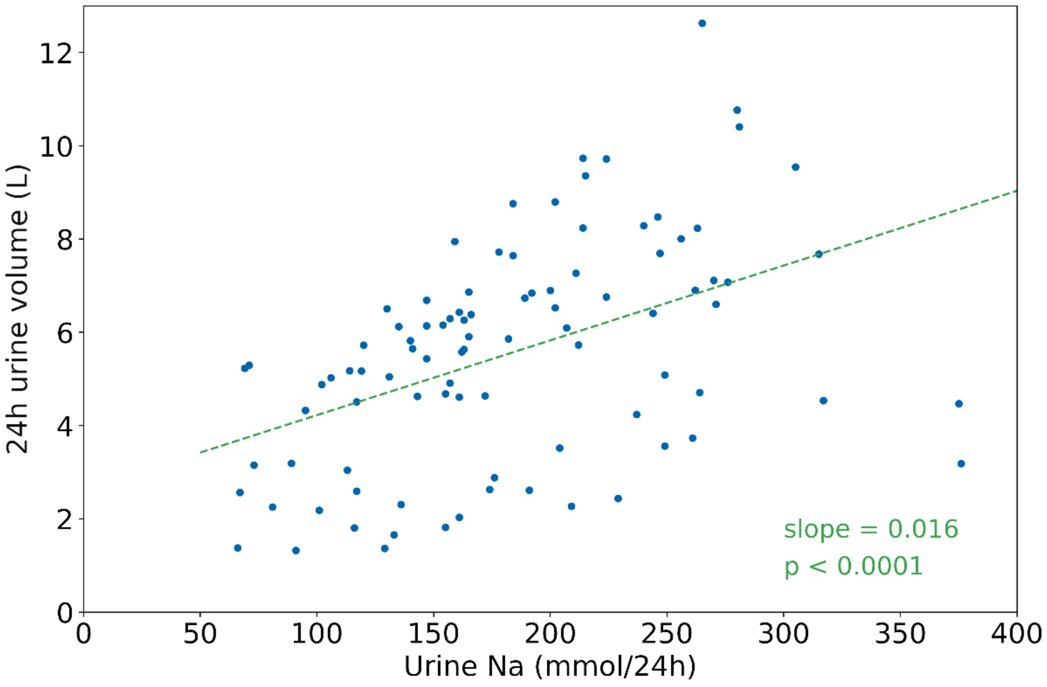

Detailed Methods

Calculation of tolvaptan usage

Data from NHS Digital was in the form of Designated Daily Dose (DDD) per quarter. DDD is a WHO-defined measure used for pharmacoeconomic data, which for tolvaptan is defined as 30mg. The amount of tolvaptan used each day in a quarter was obtained by dividing the total DDD by number of days in a standard financial quarter (90). The number of people treated was then calculated by dividing the daily tolvaptan use by the assumed average dose for ADPKD (95 or 60 mg).

Tolvaptan user survey questions

1. Do you use tolvaptan in your clinical practice?
    a. Yes
    b. No
2. Why don't you use tolvaptan for ADPKD in your clinical practice?
    a. Free text response
3. Do you use the Renal Association commentary on interpreting the NICE guideline for tolvaptan in ADPKD?
    a. Yes
    b. No
4. Why don't you use the Renal Association commentary on interpreting the NICE guideline for tolvaptan in ADPKD?
    a. Free text response
5. How do you think the guidelines could be improved? Click here to read/download the Renal Association Commentary on the Guidelines
    a. Free text response
6. Who is responsible for delivering tolvaptan treatment at your centre?
    a. Single responsible professional
    b. Multi-disciplinary team
    c. Multiple professionals functioning independently
    d. Other, comment in free text
7. What types of professionals are involved in providing tolvaptan treatment at your centre? (select all that apply)
    a. Doctor
    b. Nurse
    c. Pharmacist
    d. other, comment in free text
8. Do you perform cohort screening of ADPKD patients for tolvaptan treatment?
    a. Yes
    b. No
9. Approximately how many patients in your unit are eligible for tolvaptan therapy?
    a. <50
    b. 50-100
    c. 100-150
    d. 150-200
    e. >200

10. What proportion of your tolvaptan-eligible patients are currently on tolvaptan?
    a. 10%
    b. 25%
    c. 50%
    d. 75%
    e. >75%
    f. Information not available
11. What investigations do you use to assess patients for tolvaptan treatment? (Select all that apply)
    a. eGFR decline
    b. Ultrasound mean kidney length
    c. Baseline MRI total kidney volume
    d. Genotype
    e. Other (comment in free text)
12. Approximately what proportion of patients have declined tolvaptan treatment and why?
    a. Free text response
13. Who is responsible for the monitoring of patients at your unit on tolvaptan treatment?
    a. Primary care
    b. Secondary/tertiary care
    c. Shared care agreement between primary and secondary/tertiary care
14. Do you perform follow-up MR imaging of patients on tolvaptan treatment?
    a. Yes
    b. No
15. How else are you monitoring your tolvaptan-treated patients?
    a. Free text response
16. What proportion of tolvaptan-treated patients have stopped treatment and why?
    a. Free text response

Association between urine sodium and volume

For 24-hour urine sodium measurements below the lower limit of reporting (LLOR), values were imputed from a uniform integer distribution between (0 – LLOR). For boxplots, values below the first quartile less 1.5 times the interquartile range, or greater than the third quartile plus 1.5 times the interquartile range were considered outliers.

24-hour urine sodium (uNa_{24}) and volume were plotted using pandas and Matplotlib. The presence of a linear relationship between these values, and the disruption of this relationship at low uNa_{24} values < 50 mmol, were observed visually. Out of 117 samples, 26 samples derived from 18 patients had uNa_{24} < 50 mmol. Measured osmolality in these samples was substantially lower than in samples with uNa_{24} ≥ 50 mmol (p=0.0004), excluding the possibility of non-sodium osmolytes driving aquaresis for these patients. Most patients (78%, 14/18) who had samples with low uNa_{24}, had low uNa_{24} in less than half of their total samples, suggesting that technical errors in collection or analysis

might account for these measurements. Samples with uNa24 < 50 mmol were therefore excluded from subsequent analysis by linear regression.

Rate of eGFR decline

eGFR decline rates were determined by linear regression using the *scipy.stats.linregress* function. Baseline rate of decline was based on the 5 years preceding the first dose of tolvaptan. To account of the initial eGFR decline associated with starting tolvaptan treatment, rates calculated on treatment excluded the titration period, which was between the first dose of tolvaptan, and when a stable dose was reached. A stable dose was when there was no dose change over 2 consecutive visits.

Dates were converted to ordinal values prior to linear regression using *datetime.datetime.toordinal*. Percentage change in rate of decline was obtained through division of the rate of eGFR decline on treatment by the pre-treatment rate of eGFR decline.

The Shapiro-Wilk test strongly suggested that pre-treatment eGFR decline rate did not follow a normal distribution (p=0.0008), therefore rate of decline on treatment was compared to pre-treatment rate of decline using the Wilcoxon signed-rank test.

Mayo Class determination and rate of TKV growth

Baseline Mayo Class was calculated using an online tool (https://www.mayo.edu/research/documents/pkd-center-adpkd-classification/doc-20094754).

Rate of TKV growth was determined by linear regression of TKV measurement dates against MRI-TKV using *scipy.stats.linregress*. Dates were converted to ordinal values prior to linear regression using *datetime.datetime.toordinal*. The slope of the linear regression represented a daily rate of absolute TKV growth, which was converted to an annual rate through multiplication by 365. This was converted to percentage rate through dividing by the baseline MRI-TKV.

Relationship between baseline characteristics and treatment response

Linear regression was performed between % change in eGFR decline over 2 years and initial eGFR, eGFR drop during dose titration, initial TKV, initial spot urine osmolality, and change in urine osmolality during dose titration, using *scipy.stats.linregress*.
